# Supplementary material for: Early-branching euteleost relationships: areas of congruence between concatenation and coalescent model inferences
Source: PeerJ. 2017 Sep 8;5:e3548. doi: 10.7717/peerj.3548 (PMC5592902; doi:10.7717/peerj.3548)
Supplement: Table S1 [file peerj-05-3548-s001.docx]

**Bibliography**

Betancur-R., R., Broughton, R.E., Wiley, E.O., Carpenter, K., López, J.A., Li, C., Holcroft, N.I., Arcila, D., Sanciangco, M., Cureton II, J.C., Zhang, F., Buser, T., Campbell, M.A., Ballesteros, J.A., Roa-Varon, A., Willis, S., Borden, W.C., Rowley, T., Reneau, P.C., Hough, D.J., Lu, G., Grande, T., Arratia, G., Ortí, G., 2013. The tree of life and a new classification of bony fishes. PLoS Curr, 5, 1-33. doi:10.1371/currents.tol.53ba26640df0ccaee75bb165c8c26288

Faircloth, B.C., Sorenson, L., Santini, F., Alfaro, M.E., 2013. A phylogenomic perspective on the radiation of ray-finned fishes based upon targeted sequencing of ultraconserved elements (UCEs). PLoS ONE 8, e65923. doi:10.1371/journal.pone.0065923
