# Supplementary material for: Early-branching euteleost relationships: areas of congruence between concatenation and coalescent model inferences
Source: PeerJ. 2017 Sep 8;5:e3548. doi: 10.7717/peerj.3548 (PMC5592902; doi:10.7717/peerj.3548)
Supplement: Document S1 [file peerj-05-3548-s002.docx]

**Supplemental Document S1: Additional Phylogenetic Analyses**

*1.1 Alternative Strategies*

To further examine the stability of hypotheses we examined optimal partitioning in a Maximum Likelihood (ML) framework utilizing PartitionFinder version 1.1.1 (Lanfear et al., 2012) with RAxML version 8.2.9 (Stamatakis, 2006; Stamatakis and Ott, 2008). To verify the *BEAST result (Heled and Drummond, 2010) was a result of the coalescent model and not nucleotide evolution model choice, a concatenated tree search using the Hasegawa-Kishino-Yano (HKY) model of sequence evolution with a four-category gamma distributed rate variation (Γ) in BEAST 2 (Drummond et al., 2012) was conducted.

*1.2 Optimal Partitioning and Maximum-Likelihood Inference*

The 34-OTU dataset described in the manuscript was divided into partitions for each ultra-conserved element and PartitionFinder (Lanfear et al., 2012) was used to determine and optimal partitioning scheme. The following settings were specified with PartitionFinder: Branches were linked, only the general time reversible (GTR) model of sequence evolution with gamma distributed rate variation (Γ) od was examined, the hcluster search algorithm was specified, and the best choice was identified by the Bayesian information criterion. Four partitions were supported by PartitionFinder. The PartitionFinder configuration file and partitioning file are available in the Data Supplement. A tree search was subsequently conducted in RAxML version 8.2.9 with automatic stopping of bootstrap replicates specified (-N autoMRE) (Stamatakis, 2006; Stamatakis and Ott, 2008). The branching of early-branching euteleost lineages, and all other branchings are identical to the topology where partitioning at each locus was done (Figure 1, Supplemental Figure S1). The support at nodes for early-branching euteleosts is very high (bootstrap support > 98%).


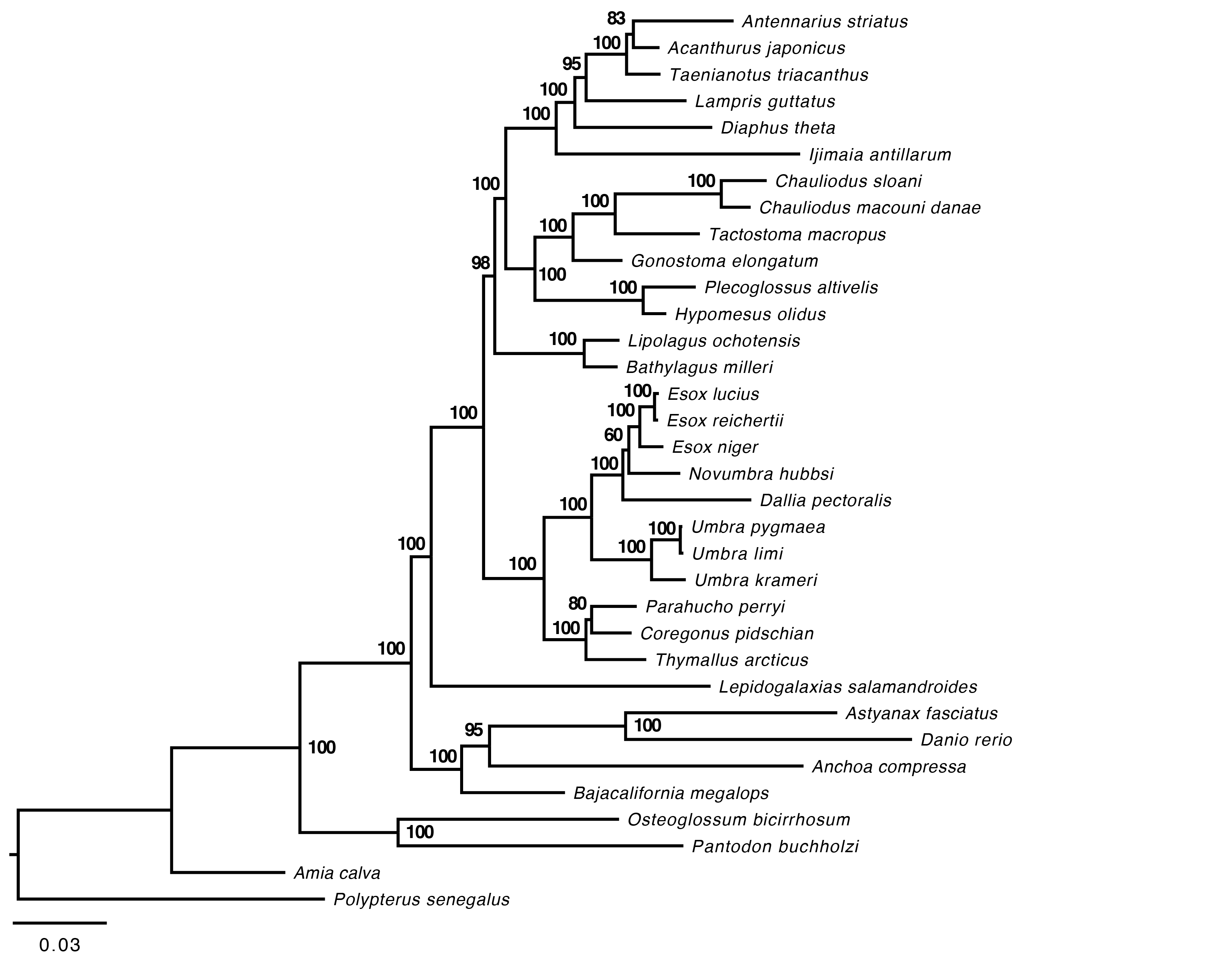


**Supplemental Figure S1 –** Phylogeny generated by an objectively determined partitioning strategy in a Maximum-Likelihood framework of the 34-OTU dataset. Four partitions were modeled with a GTR+Γ model and rate variation along with automatic stopping of bootstrap replicates. Bootstrap support values are indicated at nodes.

*1.3 Concatented Tree Search with BEAST 2*

To verify that the coalescent model, not nucleotide evolution model choice did not result in an altered topology, a concatenated tree search was conducted in BEAST 2 (Drummond et al., 2012). For each of the 53 ultra-conserved element loci in the 34 OTU dataset, the same model parameters (HKY with four category distributed rate variation and empirical base frequencies) as the *BEAST (Heled and Drummond, 2010) analysis were specified. Instead of a joint species tree and gene trees estimation, a concatenated tree search constraining each locus to the same topology was conducted. Four independent chains of 10 Million generations were combined with 10% burnin to reach effective sample sizes (ESS > 200) for all parameters. Combined, 36,004 trees were combined into a maximum clade credibility tree with a posterior cutoff of 0.5 specified. The maximum clade credibility tree with median heights is shown (Suppemental Figure S2). The resulting tree is identical to both the partitioned by ultra-conserved element and optimal partitioning RAxML trees in terms of branching patterns. Early-branching euteleost lineages receive high support (posterior probabilities = 1.00). Alternative arrangements from the other concatenated hypotheses are notable in the placement of *Bajacalifornia megalops* and the arrangements of the perciform taxa.


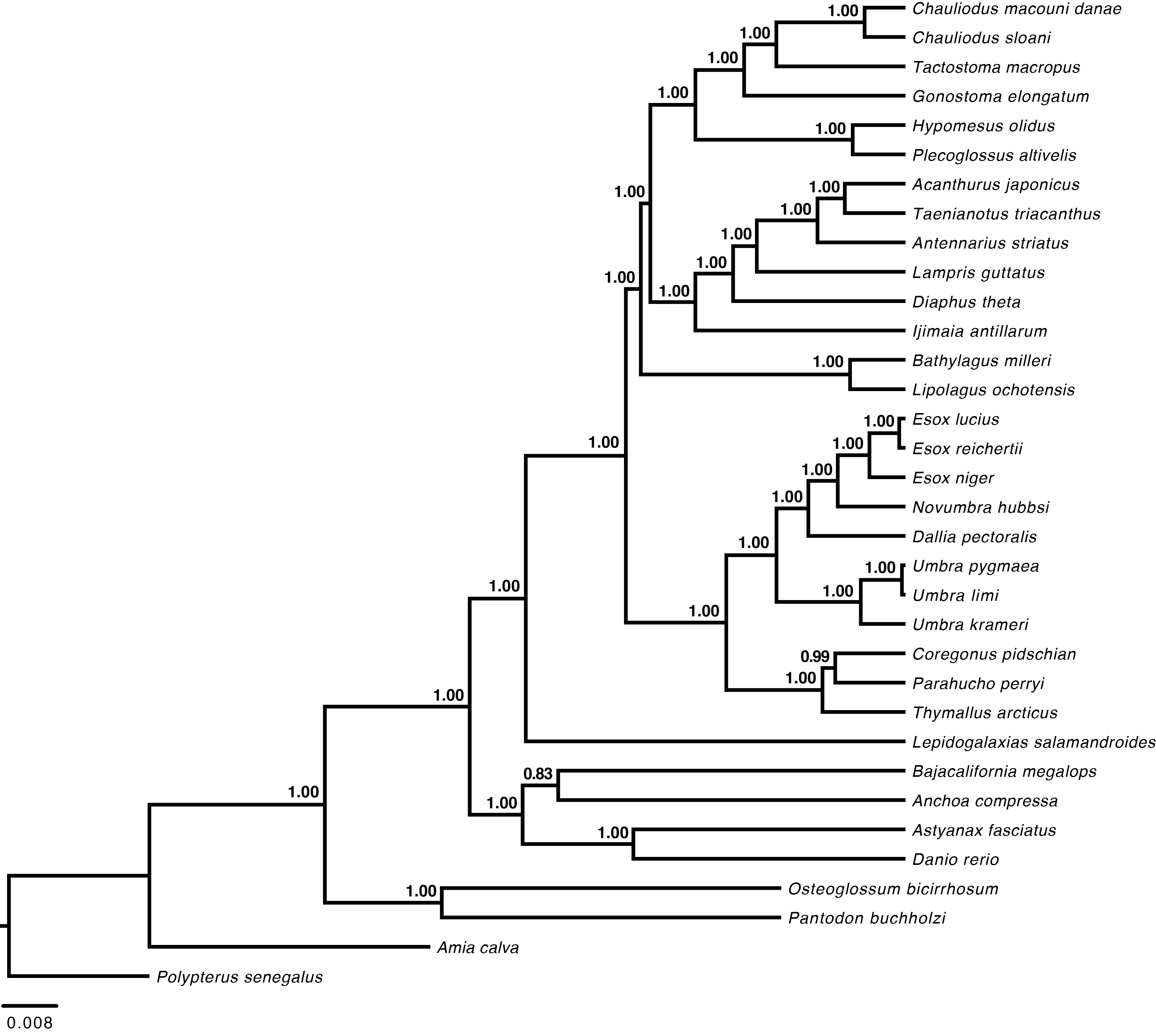


**Supplemental Figure S2 –** Concatenated tree search conducted in BEAST 2 with separate nucleotide evolution parameters of the HKY model, four category gamma distributed rate variation (Γ) and empirical base frequencies specified for each of the fifty-three ultra-conserved element loci. The maximum clade credibility tree from four separate runs for 10 Million generations is pictured after a 10% burnin. Values at nodes represent posterior probability values.

**Bibliography**

Drummond, A.J., Suchard, M.A., Xie, D., Rambaut, A., 2012. Bayesian phylogenetics with BEAUti and the BEAST 1.7. Mol. Biol. Evol. 29, 1969–73. doi:10.1093/molbev/mss075

Heled, J., Drummond, A.J., 2010. Bayesian Inference of Species Trees from Multilocus Data. Mol. Biol. Evol. 27, 570–580. doi:10.1093/molbev/msp274

Lanfear, R., Calcott, B., Ho, S.Y.W., Guindon, S., 2012. PartitionFinder: combined selection of partitioning schemes and substitution models for phylogenetic analyses. Mol. Biol. Evol. 29, 1695–1701. doi:10.1093/molbev/mss020

Stamatakis, A., 2006. RAxML-VI-HPC: maximum likelihood-based phylogenetic analyses with thousands of taxa and mixed models. Bioinformatics 22, 2688–2690. doi:10.1093/bioinformatics/btl446

Stamatakis, A., Ott, M., 2008. Efficient computation of the phylogenetic likelihood function on multi-gene alignments and multi-core architectures. Philos. Trans. R. Soc. B Biol. Sci. 363, 3977–3984. doi:10.1098/rstb.2008.0163
